# Genotype-driven therapeutic developments in Parkinson’s disease

**DOI:** 10.1186/s10020-021-00281-8

**Published:** 2021-04-19

**Authors:** Jannik Prasuhn, Norbert Brüggemann

**Affiliations:** 1 Department of Neurology, University Medical Center Schleswig-Holstein, Campus Lübeck, Lübeck, Germany; 2 Institute of Neurogenetics, University of Lübeck, Lübeck, Germany; 3 Center of Brain, Behavior and Metabolism, University of Lübeck, Lübeck, Germany

**Keywords:** Parkinson’s disease, *SNCA*, *GBA*, *LRRK2*, *PRKN (Parkin)*, *PINK1*, Translational, Monogenic, Genetic, Therapy, Treatment

## Abstract

**Background:**

Remarkable advances have been reached in the understanding of the genetic basis of Parkinson’s disease (PD), with the identification of monogenic causes (mPD) and a plethora of gene loci leading to an increased risk for idiopathic PD. The expanding knowledge and subsequent identification of genetic contributions fosters the understanding of molecular mechanisms leading to disease development and progression. Distinct pathways involved in mitochondrial dysfunction, oxidative stress, and lysosomal function have been identified and open a unique window of opportunity for individualized treatment approaches. These genetic findings have led to an imminent progress towards pathophysiology-targeted clinical trials and potentially disease-modifying treatments in the future.

**Main body of the manuscript:**

In this review article we will summarize known genetic contributors to the pathophysiology of Parkinson’s disease, the molecular mechanisms leading to disease development, and discuss challenges and opportunities in clinical trial designs.

**Conclusions:**

The future success of clinical trials in PD is mainly dependent on reliable biomarker development and extensive genetic testing to identify genetic cases. Whether genotype-dependent stratification of study participants will extend the potential application of new drugs will be one major challenge in conceptualizing clinical trials. However, the latest developments in genotype-driven treatments will pave the road to individualized pathophysiology-based therapies in the future.

## Background

Parkinson’s disease (PD) is an age-related neurodegenerative disorder characterized by the progressive degeneration of nigrostriatal dopaminergic neurons and extranigral brain regions due to the accumulation of certain α-synuclein species (Fujita et al. 2014; Moore et al. 2005; Schapira and Jenner 2011). The underlying pathophysiology is complex and involves a variety of molecular processes. Despite extensive research in unraveling the molecular mechanisms of PD, no disease-modifying treatments are available to date, and the standard patient care mainly relies on purely symptomatic therapies. Even though monogenic causes of PD (mPD) only account for a minority of PD cases, the investigation of genetic alterations sheds further light on individual disease causes and provides a unique opportunity for drug development and, subsequently, genotype-driven therapies (Blauwendraat et al. 2020). Biological findings and disease traits of mPD can also be useful to identify common disease mechanisms in idiopathic PD (IPD). Selected IPD patients may share molecular pathways with mPD which would be the pre-requisite for pathway-targeted therapies (Redenšek et al. 2017). The identification of such IPD patients requires the detection of reliable biomarkers that enable the stratification of individual IPD patients according to their underlying pathology and the development of appropriate clinical trial designs. Furthermore, the search for patients with mPD by genotyping is an important pre-requisite to gather a substantial number of potential study participants for genotype-driven treatment options and clinical trials. Genetic testing of PD patients is, however, not routinely applied in clinical practice, and consortium research efforts are needed to meet future recruitment targets. In contrast to mPD, IPD is a complex genetic disease where the interaction of genetic risk factors with environmental factors plays a pivotal role in the disease development. The individual identification of risk variants can help gain insights into particular disease pathophysiology independent of a monogenic trait. To date, three approaches have been or are presently evaluated for the stratification of patients with IPD: polygenic risk scoring, blood-based biomarkers (e.g., by measuring enzyme activity in peripheral tissue), or neuroimaging methods (e.g., by studying brain energy metabolism).

This review article will summarize the current understanding of PD genetics, challenges in the development of individualized treatments, current genotype-driven therapies under investigation, and the critical need for reliable biomarkers for patient stratification and treatment monitoring.

## Main body

### Genetics of monogenic Parkinson’s disease

PD is caused by complex interactions between genetic and environmental factors. Mutations in *SNCA* (α-synuclein*)*, *LRRK2* (Leucine Rich Repeat Kinase 2), and *VPS35* (VPS35 Retromer Complex Component) cause an autosomal dominant form of mPD whereas mutations in *PRKN* (Parkin), *PINK1* (PTEN-induced kinase 1), and *PARK7 (*oncogene DJ-1) (among others) are associated with autosomal-recessive mPD (Lill 2016). Although *SNCA* was the first identified PD-related gene almost 25 years ago it is very rare whereas mutations in *LRRK2* are the most frequent cause of mPD (Cookson 2015). The identification of the autosomal-recessive genes *PRKN*, *PINK1*, and *PARK7* linked the proposed role of mitochondrial dysfunction in the etiology of PD to genetic causes (Exnre et al. 2012). Monogenic causes are often summarized under the umbrella term of mPD even if parkinsonism is only one of the presenting symptoms and only part of a more complex or atypical phenotype (e.g., in *DNAJC6* mutation carriers, Table 1) (Puschmann 2013). Whether molecular insights of causative genes for atypical phenotypes provide translatable findings to IPD needs to be critically evaluated (Grünewald et al. 2013; Klein et al. 2007). In addition, several genes were not yet replicated in independent families or populations. Furthermore, most forms are exceedingly rare making it unlikely that specific therapies are being developed. In addition to mPD that follows the rules of Mendelian inheritance, variants in the *GBA* (Glucosylceramidase Beta) gene are an unequivocal and frequent risk factor for the development of PD and a promising future drug target.

**Table 1:** Monogenic traits of Parkinson’s disease

Gene name	Mode of inheritance	Frequency	Mutation	Proposed disease mechanisms	Predominantly involved pathway(s)	Predominant phenotype
*SNCA*	AD	Very rare	Missense or multiplication	GoF	α-Synuclein aggregation	Duplication: resembles PD Missense/triplication: LBD-like
*POLG*	AD	Rare	Missense	LoF?	Mitochondrial impairment	Atypical PD with features of mitochondriopathy
*VPS35*	AD	Very rare	Missense (up to 100%: D620N)	LoF	Endosomal/lysosomal dysfunction	Resembles PD
*DNAJC13*	AD	Very rare	Missense	LoF?	Co-chaperones and endosomal/lysosomal dysfunction	Atypical PD
*LRRK2*	AD (incomplete penetrance)	Population-dependent*	Missense (76% G2019S**)	GoF	Endosomal/lysosomal dysfunction; mitochondrial impairment	More beneficial disease course as PD
*PRKN*	AR	Rare	Missense or deletions	LoF	Mitochondrial impairment	EOPD
*PINK1*	AR	Very rare	Missense or deletions	LoF	Mitochondrial impairment	EOPD
*PARK7*	AR	Very rare	Missense or deletions	LoF	Mitochondrial impairment	EOPD
*ATP13A2*	AR	Very rare	Missense or deletions	LoF	Endosomal/lysosomal dysfunction?	Atypical PD Complex HSP
*FBXO7*	AR	Very rare	Missense	LoF	Mitochondrial impairment and endosomal/lysosomal dysfunction	Often EOPD
*PLA2G6*	AR	Very rare	Missense or deletions	LoF	Phospholipid remodeling, arachidonic acid release, leukotriene and prostaglandin synthesis, and fas‐mediated apoptosis; α-synuclein aggregation	often EOPD
*DNAJC6*	AR	Very rare	Missense or deletions	LoF	Endosomal/lysosomal dysfunction	Atypical PD
*SYNJ1*	AR	Very rare	Missense or deletions	LoF	Endosomal/lysosomal dysfunction	Atypical PD
*VPS13C*	AR	Very rare	Missense or deletions	LoF	Mitochondrial impairment	Resembles PD
*GBA*	Genetic risk factor	Common	Missense or deletions***	LoF	Endosomal/lysosomal dysfunction	More aggressive disease course as PD

The table shows independently confirmed PD genes, in which functional data is available to interpret the underlying pathomechanism. The frequency is listed as a relative measure and is based on the evaluation of the authors. The question marks depict the current level of uncertainty or unknown aspects of distinct genes

*?* unclear/uncertain, *AD* autosomal dominant, *AR* autosomal recessive, *ATP13A2* ATPase Cation Transporting 13A2, *PARK7* oncogene DJ-1, *DNAJC13* DnaJ Heat Shock Protein Family (Hsp40) Member C13, *DNAJC6* DnaJ Heat Shock Protein Family (Hsp40) Member C6, *EOPD* early-onset Parkinson’s disease, *FBXO7* F-Box Protein 7, *GBA* Glucosylceramidase Beta, *GoF* gain of function, *LoF* loss of function, *LRRK2* Leucine Rich Repeat Kinase 2, *PD* Parkinson’s disease, *PINK1* PTEN-induced kinase 1, *PLA2G6* Phospholipase A2 Group VI, *POLG* Mitochondrial Polymerase Gamma, *PRKN* Parkin, *SNCA* α-synuclein, *SYNJ1* Synaptojanin 1, *VPS13C* Vacuolar Protein Sorting 13 Homolog C, *VPS35* VPS35 Retromer Complex Component, *HSP* hereditary spastic paraplegia, *LBD* Lewy body dementia

*Approx. 20% in Ashkenazi Jews and 40% in North African Berbers (Healy et al. 2008; Lesage et al. 2006), ~ 1% in white (European or North-American ancestry) population (Heckman et al. 2013). **based on MDSGene data (https://www.mdsgene.org/d/1/g/1?action=plot&fc=0&_mu=1&_country=1 date of access: 4th of February 2021). ***Severe/Pathogenic variant: previously described in Gaucher disease, Mild/Risk factor: associated with an increased risk of PD, but not causative for Gaucher disease

### Drug targets in monogenic Parkinson’s disease

Most mPD genes and *GBA* converge to distinct molecular mechanisms and can be divided into (i) α-synuclein aggregation (Dehay et al. 2015), (ii) endosome-related involvement (Hafner Česen et al. 2012; Dehay et al. 2013; Smolders and Van Broeckhoven 2020), and (iii) those leading to mitochondrial impairment (Exnre et al. 2012). These pathways indicate potential drug targets for the disease-modifying treatment of mPD (Brüggemann and Klein 2019). Even though mPD helped identify these target pathways in the past decades, genetic stratification in clinical trials started only recently (Dehay et al. 2015; Shults et al. 2004).

### The role of α-synuclein aggregation and the SNCA gene

α-Synuclein aggregation is the pathophysiological hallmark of IPD, which has been extensively demonstrated in post-mortem studies (Moore et al. 2005; Kellie et al. 2014). Mutations in the *SNCA* gene predispose to an increased α-synuclein accumulation and aggregation as the main driver of cell-to-cell propagation of α-synuclein pathology in PD (Xu and Pu 2016). Duplications or triplications result in an increased expression of the wildtype allele with a gene dosage effect whereas point mutations have an impact on the aggregative properties of α-synuclein (Book et al. 2018). α-Synucleinopathy has not only been found in IPD and *SNCA* mutation carriers but also in other forms of mPD (Poulopoulos et al. 2012). The histopathological changes in mPD, however, are more variable and include tau pathology in some *LRRK2* carriers and the absence of α-synucleinopathy in most carriers of *PRKN* mutations (Henderson et al. 2019; Schneider and Alcalay 2017). The overall evidence of neuropathologic changes in mPD is still limited due to the rarity of autopsied cases.

Formed α-synuclein oligomers are unsuccessfully cleared by the lysosomal or ubiquitin–proteasome systems (UPS) resulting in the formation of Lewy bodies (Volpicelli-Daley et al. 2016). Rodent models demonstrated that the injection of α-synuclein fibrils into the brain activates prolonged α-synuclein aggregates and propagation in interconnected brain regions of model organisms (Luk et al. 2012; Dehay et al. 2016) and human subjects (McCann et al. 2016). The ascending propagation of α-synuclein pathology along neural structures are represented by the Braak stages, which have substantially shaped our current understanding of PD pathophysiology (McCann et al. 2016). Different target mechanisms are currently discussed to counteract the progressive propagation of α-synuclein pathology: (i) decreased α-synuclein production, (ii) decreased intracellular α-synuclein aggregation, (iii) enhanced intracellular α-synuclein degradation, (iv) enhanced extracellular α-synuclein degradation, (v) and the blockage of neural uptake of extracellular α-synuclein (Dehay et al. 2015). These target mechanisms may also expand to mPD forms with α-synuclein pathology but are thus not specific and do not interact with more upstream gene or pathway-related changes.

In animal models, the use of viral vectors successfully mediated the in-vivo production of siRNA (small-interfering RNA; double-stranded, non-coding RNA molecules, that typically lead to the targeted degradation of complementary mRNA molecules) against *SNCA* in the substantia nigra by employing numerous methods to reduce α-synuclein expression (Volpicelli-Daley et al. 2016). A decrease in α-synuclein levels could also be achieved by varying histone acetylation at the α-synuclein gene promoter and enhancer regions and by administering ß-2-adrenergic agonists (e.g., clenbuterol and salbutamol) (Mittal et al. 2017). Intrabodies bind with monomeric α-synuclein and inhibit oligomerization (Bhatt et al. 2013). Thus, in rodents with viral vector-mediated α-synuclein overexpression, an increase of intracellular α-synuclein aggregation as the cause for subsequent nigral neurodegeneration could be prevented (Volpicelli-Daley et al. 2016). The substance NPT200-11 (NPT200-11 trial, NCT02606682) was furthermore able to block the α-synuclein interface with cell membranes and slowed the oligomerization of aggregates in a mouse model (Bhatt et al. 2013). The phosphorylation of a rapamycin inhibitor was capable of promoting autophagy and reduction of α-synuclein pathology in model systems (Boyd et al. 2013). Nilotinib, a Tyrosine-protein kinase ABL1 inhibitor, was shown to inhibit protein aggregation, neurodegeneration, mitochondrial pyruvate carriers, and posttranslational modifications of α-synuclein in in mice with safety data already available for human use (PD Nilotinib, NCT02954978) (Pagan et al. 2016; Karuppagounder et al. 2014). The first preclinical trials have observed a reduction in extracellular α-synuclein or α-synuclein aggregation due to immunotherapy (Lindström et al. 2014; Tran et al. 2014; Spencer et al. 2017). The first α-synuclein immunotherapy used in a clinical PD trial was PRX002, a humanized IgG1 monoclonal antibody that acts against epitopes of the α-synuclein C-terminus (Brundin et al. 2017). An ascending-dose study in healthy volunteers proved the safety and tolerability at doses up to 30 mg/kg with a plasma half-life of 18.2 days, which was maintained for two to four weeks after a single infusion(BP39529, NCT03100149) (Schenk et al. 2017). In addition, optimized antibodies have been developed in preclinical models against different forms of mono-, oligomeric, fibrils, and aggregated forms to target different stages in α-synuclein related pathophysiology (Wang et al. 2019). Moreover, the recent development of the *SNCA* gene by antisense-oligonucleotides (ASOs; short-chain, synthetic, single-stranded oligonucleotides that bind to the complementary mRNA and modify/hinder their respective translation) has already been proven useful in in-vitro, rodent, and primate models (Uehara et al. 2019; Alarcón-Arís et al. 2018; Choong and Mochizuki 2017).

### Targeting lysosomal dysfunction in PD: the role of the GBA gene

The *GBA* gene encodes the protein glucocerebrosidase (GCase), a lysosomal hydrolase, which converts glucosylceramide to ceramide and glucose (Goker-Alpan et al. 2004; Neudorfer et al. 1996). The accumulation of undegraded substrates by compound heterozygous or homozygous *GBA* mutations has been linked to the lysosomal storage disorder Gaucher's disease (GD). Interestingly, GD patients have a higher incidence of parkinsonism, and heterozygous mutations in the *GBA* gene increase the risk of developing PD (Goker-Alpan et al. 2004). Subsequently, heterozygous *GBA* mutations are considered the most common genetic risk factor for PD, and at least one putative damaging mutation can be present in up to 10% of PD patients (Robak et al. 2017). There is a crucial delineation between ‘pathogenic’ *GBA* variants (such as those causing GD in a compound heterozygous/homozygous carrier state) and ‘PD risk factor’ *GBA* variants, which show an association with PD risk but are not considered causative for GD (Skrahina et al. 2020). Another classification of *GBA* variants embraces the distinction of ‘mild’ and ‘severe’ variants (in patients with type 1 or type 2/3 GD) (Stirnemann et al. 2017). Age at onset, phenotype and disease course of carriers of mild *GBA* variants (mGBA-PD) are comparable with IPD whereas carriers of severe *GBA* variants (sGBA-PD) have a clearly increased risk of dementia with an earlier onset and a more rapid cognitive decline (Davis et al. 2016). Disease progression of carriers of mild mutations is usually slower than in those with severe mutations but still faster than in non-carriers (Cilia et al. 2016). The GCase activity is reduced in sGBA-PD and, to a lesser extent, in mGBA-PD, whereas there is only a slight reduction in IPD (Alcalay et al. 2015). Collectively, an increase of GCase activity by targeted therapies may only be beneficial for selected IPD patients as there is currently no persuasive evidence for increased accumulation of glycosylceramides in IPD patients (Niimi et al. 2020) and no efficacy and safety data are yet available on supraphysiological GCase levels. On the other hand, several, mainly preclinical, studies have shown that GBA deficiency predisposes to α-synuclein pathology. Here, reduced GCase activity causes increased levels of ubiquitin/α-synuclein aggregates and is related to motor and cognitive problems in a rodent model (Sardi et al. 2013). An inverse relationship between GCase activity and oligomeric α-synuclein levels can be explained by a pathological feedback loop (Mazzulli et al. 2011). Changes in glycosphingolipid homeostasis can affect the membrane composition and impair lysosomal function and vesicular transport, thus enhancing α-synuclein aggregation. This process results in selective synaptic dysfunction and neuronal degeneration (Schapira 2015).

Different treatment options are discussed in GBA-PD but also selected patients with IPD: (i) substrate reduction (as often considered for the treatment of GD), (ii) external GCase augmentation, and (iii) the enhancement of GCase activity (e.g., by ambroxol). Substrate reduction can be achieved by different mechanisms: Glucosylceramide synthase inhibitors decrease glycosphingolipid levels and are used to treat the hematological and visceral presentations of GD patients. The clinical trial GZ/SAR402671 (NCT02906020) showed that the glucosylceramide synthase inhibitor venglustat sufficiently crossed the blood brain barrier (BBB) in humans (Davis et al. 2016). In mouse models of GD-related synucleinopathy and alpha-synuclein overexpression, venglustat led to a decrease in alpha-synuclein expression (Mazzulli et al. 2011). Whether this approach may also be suitable for GBA-PD is currently under investigation in a clinical phase II trial in patients (Moves PD, NCT02906020).

The external augmentation of GCase in the brain requires the penetration of the enzyme through the blood brain barrier which cannot be sufficiently achieved with enzymes used in GD treatment (Sun et al. 2020). One approach is therefore a gene therapy-mediated viral overexpression of exogenous GCase in the brain that was shown to reverse behavioral and pathological abnormalities by restoring the membrane glycosphingolipid balance (Sardi et al. 2011; Rockenstein et al. 2016). This observation supports gene therapy aiming to increase GCase levels in the brain. In keeping, adeno-associated viruses are safe and biologically active vectors that target GCase augmentation, reverse cognitive problems, and reduce α-synuclein in an *SNCA*^*A53T*^ mouse model (Hafner Česen et al. 2012). Moreover, a previous investigation showed that increased lysosomal GCase activity could be achieved by optimizing the delivery route, vector subtypes, small molecules, small molecular chaperones, and brain distribution in critical brain regions (Gegg and Schapira 2018). The third proposed approach for *GBA*-targeted therapies is the enhancement of GCase activity, e.g., by the repurposed drug ambroxol in order to enhance GCase activity and to reduce α-synuclein and S129-phosphorylated α-synuclein protein levels as shown in nonhuman primates. At present, the clinical trials UCL 15/0118 (NCT02941822) and R15-006 (NCT02914366) tested the safety, efficacy, and tolerability of ambroxol in PD (Migdalska-Richards et al. 2016, 2017). Preliminary data of human subjects are already available, supporting sufficient BBB crossing and molecular target site enrichment (Mullin et al. 2020). Another investigational drug class are noninhibitory GCase chaperones such as NCGC758 and NCGC607 that can bind to GCase at the active site and lead to conformational changes that enhance GCase activity. These chaperones penetrate the brain, increase lysosomal activity, GCase translocation to lysosomes, and reduce substrate and α-synuclein accumulation (Dehay et al. 2013). Another clinical trial of afegostat tartrate in GD (AT2101, NCT00433147) showed increased GCase activity and enzyme stabilization but did not show significant clinical improvement in GD patients, and the trial was discontinued (Boyd et al. 2013).

### Challenges and opportunities of LRRK2-targeted clinical trials

The first gene mutation in *LRRK2* was identified in a family series of autosomal-dominant parkinsonism (Paisán-Ruíz et al. 2004). The *LRRK2*^*G2019S*^ accounts for the vast majority of *LRRK2*-associated PD worldwide and is highly frequent in certain populations of PD patients, e.g. in Israel and North Africa (Trinh et al. 2018). Symptomatic *LRRK2*^*G2019S*^ mutation carrier usually present with a postural instability and gait difficulty (PIGD) phenotype, but show a relatively mild cognitive and motor decline during the overall disease course (Saunders-Pullman et al. 2018). Together with other *LRRK2* variants, the *LRRK2*^*G2019S*^ result in a gain of function (GOF) with an increase of LRRK2 kinase activity. The LRRK2 protein has a complex multidomain structure and belongs to the family of protein kinases, which play a fundamental role in the control and regulation of complex cellular processes by transferring phosphate groups to target proteins. The kinase domain of LRRK2 shares similarities with mitogen-activated protein (MAP) kinases, which play a central role in mediating cellular stress. Even though the precise mechanism of LRRK2 is poorly understood, the disease-causing GOF mutations allows heuristic treatments by inhibition of its activity. Currently, two main strategies exist for *LRRK2*-targeted treatment strategies: (i) pharmacological inhibition of LRRK2 activity and (ii) silencing of the *LRRK2* gene by the use of ASOs. Both options aim to reduce LRRK2 activity in the CNS whereby ASOs may bypass potential peripheral adverse effects of kinase inhibitors due to its intrathecal application (Cookson 2015). DNL201, a small molecule LRRK2 inhibitor, reduced LRRK2 activity levels by more than 90% in a phase I study in healthy volunteers (DNLI-B-0001, NCT04551534). Ras-related protein Rab10 substrate phosphorylation and *LRRK2* S935 phosphorylation were used to measure treatment response (by a decrease in peripheral LRRK2 activity) in blood. A trial with DNL151, a second LRRK2 inhibitor, is still actively recruiting healthy volunteers (DNLI-C-0001, NCT04557800) (Zeuner et al. 2019). Preclinical data suggested that inhibition of LRRK2 could be associated with pulmonary morphological changes in nonhuman primates, resulting in potential safety concerns. Here, targeting LRRK2 with a high dose of three compounds resulted in accumulating lamellar bodies in type-II pneumocytes (Fuji et al. 2015). However, these morphological abnormalities could be reversed after two weeks off dose, and no pulmonary abnormalities were found at the highest doses. Furthermore, there was no association of loss of function variants with a putatively decreased LRRK2 kinase activity and a specific phenotype or disease state in human databases (Whiffin et al. 2020).

### Improving mitochondrial bioenergetics and antioxidative treatment strategies: PRKN and PINK1

Mitochondrial dysfunction is one of the main concepts in PD pathogenesis. Mitochondria play a fundamental role for a plethora of cellular processes relevant for the supply of energy, the overall cellular homeostasis, and neuronal survival. The first evidence for a role in PD derived from environmental studies illustrating the effect of neurotoxic agents in inhibiting the mitochondria's electron transport chain (ETC) (Schapira and Jenner 2011). The discovery of the autosomal recessively inherited genes *PRKN* and *PINK1* provided further evidence to support a strong contribution of mitochondrial dysfunction to PD (Exnre et al. 2012). Under physiological conditions, PINK1 recruits Parkin to damaged mitochondria and leads to the clearance of mitochondria via the UPS, a process referred to as mitophagy (Park et al. 2018). Parkin and PINK1, therefore, jointly serve as a molecular quality control system (McWilliams and Muqit 2017). Possible therapeutic or preventive approaches in carriers of *PRKN* or *PINK1* mutations are thus (i) the enhancement of *PRKN* or *PINK1* expression, (ii) the prevention of Parkin or PINK1 inactivation, and (iii) the control of the downstream Parkin/PINK1 signaling pathway (Gaki and Papavassiliou 2014). However, mitochondrial dysfunction extends to a variety of pathological mechanisms including impaired mitochondrial biogenesis, fusion and fission functions, trafficking, metal ion and calcium homeostasis, neuroinflammation, and pro-apoptotic signaling (Dextera and Jenner 2013). These different aspects may be suitable as potential treatment targets. Alternative strategies for genotype-driven therapies in mitochondrial dysfunction consists of the enhanced clearance of dysfunctional mitochondria via mitophagy or other mitochondrial stress response pathways (Aman et al. 2020), the improvement of mitochondrial biogenesis (e. g., by glucagon-like peptide 1 [GLP1] receptor agonist exenatide exposition) (Athauda et al. 2017), gene therapies targeting the mitochondrial or nuclear genome (Choong and Mochizuki 2017), addressing mitochondrial calcium and metal ion dyshomeostasis (e.g., by iron chelators) (Sun et al. 2018; Rani and Mondal 2020), targeting the intersection to neuroinflammation (e.g., by disruption of interleukin 6 [IL-6] signaling) (Borsche et al. 2020), and stem cell therapies (Cheng et al. 2020). However, bioenergetic depletion and increased reactive oxygen species [ROS] production are common to all types of mitochondrial dysfunction, they do most likely recapitulate one of the earliest pathophysiological events not only in mPD but also IPD, and were thus the primary target mechanisms for most of the recent studies addressing mitochondrial pathology (Prasuhn et al. 2021). The most extensively investigated compound has been coenzyme Q10, a mitochondrial enhancer that, however, failed to show efficacy in most studies (see Table 2). One potential explanation for the negative outcome is the lack of genetic stratification of PD patients to enrich for patients with a strong contribution of mitochondrial dysfunction (e.g., biallellic *PRKN* or *PINK1* mutation carriers) as listed in Table 2. To our knowledge, only two clinical trials (MitoPD [DRKS00015880] and PD-K2 [DRKS00019932]) are actively recruiting to date that use a combination of genetic stratification and treatment-response monitoring by neuroimaging (Prasuhn et al. 2019; Prasuhn et al. 2021). In the MitoPD study, groups are defined by a varying degree of predicted mitochondrial dysfunction: homozygous/compound heterozygous *PRKN/PINK1* mutation carriers, heterozygous *PRKN/PINK1* mutation carriers, and two IPD groups defined by the statistical extrema as determined by a mitochondrial PRS (Prasuhn et al. 2019). In PD-K2, homozygous/compound heterozygous *PRKN/PINK1* mutation carriers, IPD patients, and healthy controls are included (DRKS00019932). Both studies have multimodal neuroimaging in common that will be applied as a surrogate marker for examining beneficial effects of in-vivo brain energy metabolism, i.e., by determining the change of energy equivalents using ^31^Phosphorus Magnetic Resonance Spectroscopy Imaging (^31^P-MRSI).

**Table 2:** Active clinical trials targeting distinct pathways in Parkinson’s disease patients

Trial name	Registration number	Study design	Outcomes	Study participants
α*-Synuclein aggregation*				
Impact of Bosutinib on safety, tolerability, biomarkers and clinical outcomes in dementia with lewy bodies	NCT03888222	MonoC, DB, R, PC	1st: Safety and tolerability	DLB patients
Single ascending dose study of MEDI1341 in healthy volunteers	NCT03272165	MultiC, DB, R, PC	1st: Safety and tolerability	HVs
*Endosomal/lysosomal dysfunction*				
A study to evaluate the safety, tolerability, and pharmacokinetics of BIIB094 in adults with Parkinson's disease (REASON)	NCT03976349	MultiC, DB, R, PC	1st: Safety and tolerability	mPD (*LRRK2*), IPD patients
A study to evaluate the safety, tolerability, pharmacokinetics, and pharmacodynamics of DNL151 in healthy volunteers	NCT04557800	MultiC, DB, R, PC	1st: Safety and tolerability	HVs
Study to evaluate DNL151 in subjects with Parkinson's disease	NCT04056689	MultiC, DB, R, PC	1st: Safety and tolerability	PD patients
Study to evaluate DNL201 in subjects with Parkinson's disease	NCT03710707	MultiC, DB, R, PC	1st: Safety and tolerability	mPD (*LRRK2*), IPD patients
Phase 1/2a clinical trial of PR001A in patients with Parkinson's disease with at least one GBA1 mutation (PROPEL)	NCT04127578	MultiC, DB, R, PC	1st: Safety and tolerability	mPD (*GBA*), IPD patients
Ambroxol as a treatment for Parkinson's disease dementia	NCT02914366	MonoC, DB, R, PC	1st: ADAS-cog and ADCS-CGIC	PDD patients
*Mitochondrial dysfunction*				
Ursodeoxycholic acid as a novel disease-modifying treatment for Parkinson's disease	NCT03840005	MultiC, DB, R, PC	1st: Safety and tolerability 2nd: various	IPD patients
An omics-based strategy using coenzyme Q10 in patients with Parkinson’s disease	DRKS00015880	MultiC, DB, R, PC	1st: MDS-UPDRS-III 2nd: ^31^P-MRSI	mPD (*Parkin* and *PINK1*) and genetically stratified IPD patients
Nicotinamide supplementation in early Parkinson's disease (NOPARK)	NCT03568968	MultiC, DB, R, PC	1st: MDS-UPDRS-III	Treatment naïve PD patients
Metabolic Cofactor Supplementation in Alzheimer's disease (AD) and Parkinson's disease (PD) patients	NCT04044131	MultiC, DB, R, PC	1st: assessment of cognition, ADL, and MDS-UPDRS-III	IPD or AD patients
The use of vitamin K2 in patients with Parkinson's disease and mitochondrial dysfunction (PD-K2)	DRKS00019932	MonoC, DB, R, PC	1st: ^31^P-MRSI 2nd: additional neuroimaging marker to assess mitochondrial dysfunction	mPD (*Parkin* and *PINK1*), IPD patients, and HVs

In this table, active clinical trials are listed that target either α-synuclein aggregation, endosomal/lysosomal dysfunction, or mitochondrial impairment. The registration numbers are derived from clinicaltrials.gov or the German Clinical Trials Register (accessed: 12th of December 2020). 1st: primary endpoint. 2nd: secondary endpoint

^*31*^*P-MRSI* ^31^Phosphorus Magnetic Resonance Spectroscopy Imaging, *ADAS-cog* Alzheimer’s Disease Assessment Scale-Cognitive Subscale, *ADCS-CGIC* Alzheimer’s Disease Cooperative Study—Clinical Global Impression of Change, *ADL* activites of daily living, *DB* double-blind, *DLB* Lewy Body Dementia, *PDD* Parkinson’s disease dementia, *GBA* Glucosylceramidase Beta, *HV* healthy volunteers, *IPD* idiopathic Parkinson’s disease, *LRRK2* Leucine Rich Repeat Kinase 2, *MDS-UPDRS-III* Movement Disorders Society Unified Parkinson’s Disease Rating Scale Subscore III, *MonoC* monocentric, *mPD* monogenic Parkinson’s disease, *MultiC* multicentric, *PC* placebo-control, *PD* Parkinson’s disease, *PDD* Parkinson’s disease dementia, *PINK1* PTEN-induced kinase 1, *R* randomized

### The interconnectedness of pathophysiological pathways in monogenic PD: one therapy, many targets

One key motivation for the study of mPD is the translation of molecular insights into the pathogenesis of IPD (Lin and Farrer 2014). The idea that molecular processes can be pinpointed down towards a single gene variant may, however, be a substantial oversimplification (Blauwendraat et al. 2020) as molecular processes are interwoven and complex, also in mPD. Based on our current understanding of PD pathophysiology, α-synuclein aggregation is the primary disease mechanism present in IPD, GBA-PD, and many but not all mPD cases. The histopathological findings in mPD are more heterogeneous and include tau pathology in *LRRK2* and the absence of α-synuclein deposition in most autopsied *PRKN* mutation carriers. As mentioned above, α-synuclein reduction is a main target for IPD. Thus, mPD patients who also exhibit relevant α-synuclein pathology, most importantly GBA-PD, should also benefit from this pathophysiologically based therapy. For the other forms of mPD, the effect of these therapies is less predictable. It is, therefore, necessary to establish reliable biomarkers, e.g. α-synuclein PET, to sufficiently stratify PD patients largely independent of their genotype.

The enhancement of GCase leads to a reduction in α-synuclein pathology and may be a target of interest for other α-synuclein-related forms of mPD and the majority of IPD patients (Schapira 2015; Gan-Or et al. 2017). Recent developments of selective inhibitors of glycosphingolipid biosynthesis and noninhibitory pharmacological chaperones of glycosphingolipid processing enzymes are therefore promising treatment approaches. Current limitations with respect to BBB penetration or off-target-effects are limiting their clinical usability (Sybertz and Krainc 2014).

**Fig. 1:**
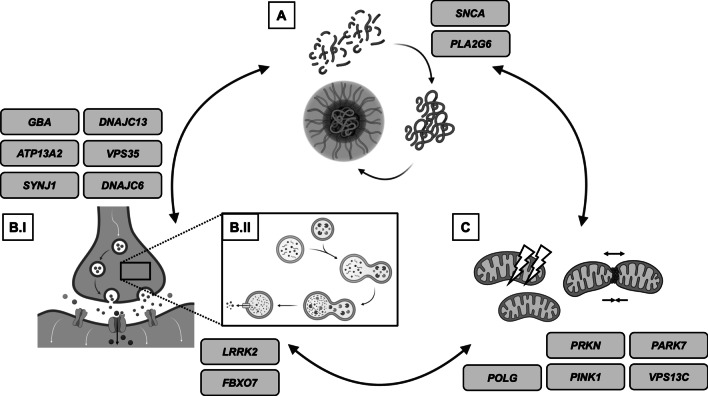
Converging pathways in Parkinson’s disease pathophysiology and relevant genes associated. The main disease mechanisms and current drug targets for mPD and GBA-PD are summarized. The links between key pathophysiological aspects are highlighted with double arrows indicating that translational therapies targeting related pathways may also be of use for a plethora of mPD, GBA-PD and IPD cases. Panel **A** depicts the aggregation of monomeric to oligomeric α-synuclein aggregates resulting in the formation of Lewy's bodies. Panel **B.I** and **B.II** symbolize endosomal disturbances, resulting in impaired neurotransmitter release (in particular VPS35) or impaired degradation of complex molecule structures by autophagy. Panel **C** illustrates mitochondrial damage, e.g., caused by oxidative stress (highlighted with thunderbolts), which can result in impaired mitochondrial dynamics (fusion and fission processes) among other downstream effects. ATP13A2: ATPase Cation Transporting 13A2. DNAJC13: DnaJ Heat Shock Protein Family (Hsp40) Member C13. DNAJC6: DnaJ Heat Shock Protein Family (Hsp40) Member C6. FBXO7: F-Box only protein 7. GBA: Glucosylceramidase Beta. GBA-PD: GBA-associated Parkinson’s disease. LRRK2: Leucine Rich Repeat Kinase 2. mPD: monogenic Parkinson’s disease. PARK7: oncogene DJ-1. PINK1: PTEN-induced kinase 1. PLA2G6: Phospholipase A2 Group VI. POLG: Mitochondrial Polymerase Gamma. PRKN: Parkin. SNCA: α-synuclein. SYNJ1: Synaptojanin 1. VPS13C: Vacuolar Protein Sorting 13 Homolog C. VPS35: VPS35 Retromer Complex Component

Third, there is supporting evidence that almost all mPD-causing gene variants are somewhat related to an impairment of mitochondrial function (Shadrina et al. 2010). Whether these patient groups may benefit from mitochondrial enhancers is still under debate. Figure 1 provides an overview of the interconnected character of highlighted genes in mPD.

Fourth, evidence from epidemiological (Gao and Chen 2011), neuroimaging (Wilson et al. 2019a), post-mortem (McGeer et al. 1988), and preclinical studies (Lindestam Arlehamn et al. 2020) suggest that neuroinflammation may be a shared pathophysiological hallmark of PD etiology. Epidemiological evidence points towards potential benefits of non-steroidal anti-inflammatory drugs, but published meta-analyses yield conflicting results (Bornebroek et al. 2007; Gagne and Power 2010; Samii et al. 2009). The long-term use of TNFα-targeted antibodies in patients with inflammatory bowel disease leads to a significant reduction in PD risk (Peter et al. 2018). The interconnectedness of the proposed pathomechanisms of PD (α-synuclein aggregation, endosome-related pathologies, and mitochondrial impairment) to neuroinflammation has been demonstrated for α-synuclein pathology (Li et al. 2019), increased LRRK2 activity (Lee et al. 2017), GCase alterations (Sanyal et al. 2020), and *PRKN/PINK1*-related mitochondrial dysfunction (Borsche et al. 2020). Therefore, targeting neuroinflammation could provide another substantial treatment opportunity for PD. However, the precise cellular and humoral driver of neuroinflammation in PD is still unclear, limiting yet the translation to clinical trials (Hirsch and Standaert 2020).

The ongoing discovery of disease mechanisms will possibly result in a combination of tailored disease-modifying therapies for individual PD patients. This will be perhaps somehow comparable with the symptomatic treatment of PD patients where the optimized combination of anti-Parkinsonian drugs is used to treat the individual disease burden of a given patient.

### The role of targeted therapies in idiopathic PD patients

The potential causes of lacking disease modification in PD are manifold and include too advanced neurodegeneration, insufficient target engagement, a varying contribution of individual disease mechanisms across IPD patients and a too short observation interval. The enrichment of study cohorts by genetically well-defined participants is crucial for developing targeted therapies, and genotype-driven therapies are currently under investigation (see Table 2). Advancements in the genetic screening of PD patients have shown that mPD and GBA-PD cases may be more frequent than previously suspected (Skrahina et al. 2020). Genetic testing is, however, still not routinely applied in the diagnostic workup or clinical trial recruitment (Billingsley et al. 2018). This now becomes increasingly relevant due to the recent progress in translational therapies that should not be withheld from individuals with an unknown but potentially treatment-qualifying genotype. Genetic testing should be considered early in PD patients' diagnostic management to meet the narrowing window of opportunity for disease-modifying treatments. In other fields, e.g., cancer treatment, the consideration of genetic variants has already entered clinical practice and led to the development of more efficient adaptive clinical trial designs (Li et al. 2007; Chow and Chang 2008; Berry 2012). Genetic testing will increasingly become important for the therapeutic management of neurological patients. As an example, the recently FDA-approved oligonucleotide drug Nusinersen requires genetic testing of patients with spinal muscular atrophy to clarify the genetic diagnosis and to evaluate patients for their eligibility to participate in clinical trials (Chiriboga 2017). The development of trial designs for neuroprotective treatments itself faces significant challenges: Long interventional periods are needed to demonstrate the disease-modifying effects of investigational drugs. While considering the long-lasting prodromal phase of PD, it is desirable to identify not yet diseased individuals for neuroprotective therapies (Heinzel et al. 2019). However, there is currently a gap of knowledge concerning one individual's conversion to PD.

To extend the promising approach of targeted therapies in mPD to IPD cases, biomarkers are needed to group patients based on their underlying disease etiology (e.g., identifying those with critical mitochondrial impairment). The disease-modifying management of PD is herewith more challenging due to the absence of validated and dynamic mechanism-based biomarkers.

### Need for reliable (para-)clinical biomarkers in the design of clinical trials for disease-modifying approaches

Biomarkers in clinical PD trials are mainly required to demonstrate target engagement and to quantify disease progression. Stratification of PD patients based on their primarily involved disease mechanisms is one substantial prerequisite for targeted therapies (Redenšek et al. 2017). Two main concepts are of importance: state and trait biomarkers. State biomarkers often refer to the genetic background of one's individual. This also includes monogenic gene variants (mPD) and the complex genetic architecture of IPD patients (e.g., by PRS referring to a predominant disease mechanism) (Heinzel et al. 2019). Trait biomarkers should recapitulate the pathophysiological processes caused by the aforementioned genetic variants and be responsive to interventions. To date, two main concepts for dynamic biomarkers are under evaluation: biomarkers based on peripheral tissues (e.g., blood or CSF based assays) and neuroimaging methods (Burciu et al. 2017; Postuma and Berg 2016; Bloem et al. 2019; Parnetti et al. 2019). There is one general concern about biomarkers of peripheral tissues: The negligible biomass of affected brain regions compared to the human body's remaining tissue requires hypersensitive analytical methods and can still be overshadowed by physiological background noise (Davis et al. 2020). Peripheral biomarkers for the converging pathophysiological mechanisms (i) α-synuclein aggregation, (ii) endosome-related, and (iii) mitochondrial impairment have already been evaluated and yielded contradictory results (Parnetti et al. 2019; Sharma et al. 2013). In most reports, their capability to treatment responses has not yet been evaluated. Neuroimaging offers the opportunity for mostly non-invasive analyses of affected brain tissue. Neuroimaging studies performed on *SNCA *(Si et al. 2019), *GBA *(Greuel et al. 2020), *LRRK2 *(Simuni et al. 2020), and *PRKN/PINK1 *(Van Nuenen et al. 2009; Anders et al. 2012; Nuenen et al. 2009) have been successfully used to illustrate neuroanatomical and functional group differences. These studies include brain mapping of the serotoninergic system in (pre-)symptomatic *SNCA*^*A53T*^ mutation carrier (Wilson et al. 2019), or PET studies investigating the serotoninergic, dopaminergic, and cholinergic neurotransmitter systems in (pre-)symptomatic *LRRK2* mutation carriers (Wile et al. 2017; Liu et al. 2018). In addition, a recent study has shown that brain metabolic networks in *GBA* or *LRRK2* mutation carrier showed a distinct network pattern (Schindlbeck et al. 2020). In summary, these studies suggest that the plethora of specific neuroimaging methods can illustrate genotype-specific brain changes and can also be applied to investigate pre-manifest mutation carriers. The latter is of high translational relevance as these neuroimaging biomarkers will open a unique window of opportunity for pre-manifest, targeted, and neuroprotective treatment strategies. Even though these promising results on genetically defined PD patients, the used methods often lack specificity with respect to the potentially treatable disease mechanism. Consequently, imaging studies are often limited to the analysis of neurodegenerative changes and do not sufficiently take disease biology into account. One notable exception is the study of brain energy metabolism for the characterization of mitochondrial dysfunction. For example, the PET tracer [^18^F]BCPP‐EF has been used to investigate Complex I dysfunction in PD patients (Wilson et al. 2020). Non-invasive magnetic resonance spectroscopy imaging (MRSI) allows the in-vivo measurement of Lactate (^1^H-MRSI) and high energy phosphates such as ATP levels (^31^P-MRSI) (Bonvento et al. 2017). These methodologies have also been advanced to allow for dynamic measurements (such as ATP synthesis rate) (Clifford et al. 2020). In addition, the combination of functional MRI (by measuring the blood-oxygen-level-dependent signal, BOLD) and arterial spin labeling (ASL) can be used to study the cerebral oxygen consumption rate (Germuska et al. 2019), and near-infrared based spectroscopy is capable to quantify the redox state of Cytochrome c (Holper et al. 2019). The availability of these methods is restricted by different hardware setups and methodological limitations. Also, intra- and intersite reliability needs to be ensured and critically assessed before being applied in clinical trials. In summary,

## Conclusion

The genetic discoveries in PD have aided the deepened understanding of clinical manifestations, underlying pathogenesis, and the potential for targeted therapies (Brüggemann and Klein 2019). Even though our current understanding of disease biology is continuously expanding, existing knowledge gaps need to be addressed in the future. Reliable biomarkers are needed that specifically recapitulate pathophysiological hallmarks for patient stratification and the monitoring of treatment responses. Genetic testing in 'idiopathic' or 'sporadic' PD patients is the prerequisite to identify individuals for genotype-driven therapies. Genotype-dependent stratification of study participants will extend the potential application of targeted drugs. Biomarker-assisted clinical trials will substantially benefit from new adaptive designs. However, the latest developments in genotype-driven treatments will, in the midterm, hopefully provide substantial benefits for PD patients and result in the first disease-modifying therapies.

## Data Availability

Not applicable.

## Abbreviations

^1^H-MRSI: Proton magnetic resonance spectroscopy imaging
^31^P-MRSI: ^31^Phosphorus resonance spectroscopy imaging
ASL: Arterial spin labelling
ASO: Anti-sense oligonucleotides
BBB: Blood brain barrier
BOLD: Blood-oxygen-level-dependent
DNAJC6: DnaJ heat shock protein family (Hsp40) member C6
ETC: Electron transport chain
GBA-PD: GBA-associated Parkinson’s disease
GBA: Glucosylceramidase Beta
GCase: Glucosylceramidase
GD: Gaucher’s disease
GLP1: Glucagon-like peptide 1
GOF: Gain of function
GWAS: Genome-wide association studies
IgG1: Immunoglobulin G type 1
IL-6: Interleukin 6
IPD: Idiopathic Parkinson’s disease
LRRK2: Leucine rich repeat kinase 2
MAP: Mitogen-activated protein
mGBA-PD: Carrier of a mild GBA variant with associated Parkinson’s disease
mPD: Monogenic Parkinson’s disease
MRI: Magnetic resonance imaging
PD: Parkinson’s disease
PARK7: Oncogene DJ-1
PET: Positron emission tomography
PINK1: PTEN-induced kinase 1
PRKN: Parkin
PRS: Polygenic Risk Score
ROS: Reactive oxygen species
sGBA-PD: Carrier of a severe GBA variant with associated Parkinson’s disease
siRNA: Small interfering ribonucleic acid
SNCA: α-Synuclein
TNFα: Tumor necrosis factor alpha
UPS: Ubiquitin–proteasome system
VPS35: VPS35 retromer complex component
